# Peptide Nucleic Acid Probes for Pretargeted Affibody-Mediated Delivery of Cytotoxic Payloads to HER2-Positive Carcinoma

**DOI:** 10.1021/acs.bioconjchem.6c00035

**Published:** 2026-07-30

**Authors:** Jacob Clinton, Kristina Westerlund, Astrid Haraldsson, Ebba Nordén, Tilde Hagelin, Eleftherios Papalanis, Moeen Ud-din, Anzhelika Vorobyeva, Torbjörn Gräslund, Amelie Eriksson Karlström, Maryam Oroujeni

**Affiliations:** † Department of Protein Science, School of Engineering Sciences in Chemistry, Biotechnology and Health, 7655 KTH Royal Institute of Technology, Stockholm 10691, Sweden; ‡ Department of Immunology, Genetics and Pathology, 8097 Uppsala University, Uppsala 75185, Sweden

## Abstract

This study investigates how the structural design of secondary PNA probes affects their ability to carry the cytotoxic agent DM1 in a novel affibody-PNA-based pretargeting approach directed at Human Epidermal growth factor Receptor 2 (HER2)-expressing cells. Six different DM1-conjugated secondary 8-mer PNA probes with strategic hydrophilic modifications were designed and evaluated. Surface plasmon resonance analysis confirmed that the modifications preserved hybridization to the HER2-targeting affibody-PNA primary probe, with all secondary probes showing similar binding kinetics (*K*_D_ = 310–660 pM). Biodistribution studies in nontumor-bearing NMRI mice with lutetium-177 (^177^Lu)-labeled secondary probes revealed that probe modifications dramatically influenced organ distribution patterns. The secondary probes SP2, SP5, and SPc demonstrated favorable biodistribution profiles with reduced accumulation in critical organs, i.e., liver and kidneys. Receptor-mediated endocytosis was also affected by secondary probe design, showing an increase in cellular internalization for SP5 (20.4% after 24 h) compared to SP2 (12.1%). Our findings highlight how rational design can optimize in vivo pharmacokinetics while preserving binding properties essential for effective pretargeting, providing a foundation for developing novel targeted cytotoxic delivery systems with potentially improved therapeutic indexes.

## Graphical abstract

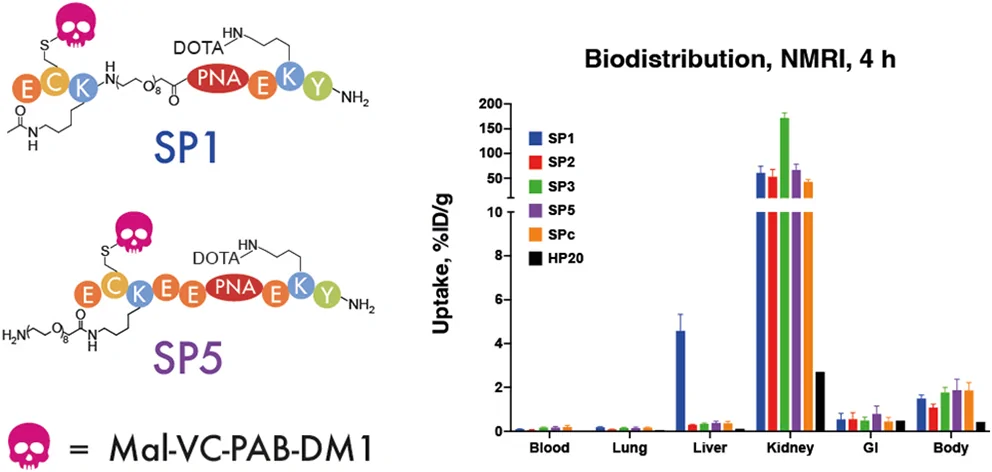

## Introduction

Targeted cancer therapies aim to deliver more effective treatment with fewer side effects compared to conventional systemic approaches. Among these, antibody-drug conjugates (ADCs) have shown remarkable success by combining antibody specificity with potent cytotoxic agents. ADCs consist of three key components: a monoclonal antibody (mAb) for tumor targeting, a cytotoxic payload for cell killing, and a specialized linker that maintains stability in circulation but enables drug release inside target cells. Currently, 15 ADCs are approved by the United States Food and Drug Administration (FDA), and more than 100 ADCs are under clinical development.^1^

In 2013, trastuzumab emtansine (T-DM1, Kadcyla) became the first ADC to be FDA-approved for solid tumors, specifically as second-line therapy for metastatic Human Epidermal growth factor Receptor 2 (HER2)-positive breast cancer. HER2 is a well-validated therapeutic target, overexpressed in approximately 20% of breast and gastric cancers and 3% of colorectal malignancies.^2^ T-DM1 is an antibody-maytansinoid conjugate (AMC) that combines the anti-HER2 antibody trastuzumab with the cytotoxic maytansine derivative DM1 via a noncleavable linker. Maytansinoids are potent antimitotic drugs that work by inhibiting tubulin polymerization, halting proliferation, and inducing apoptosis in cancer cells.^3^ Despite its clinical impact, hepatotoxicity has emerged as a significant concern when treating patients with T-DM1. In large clinical trials, between 20–80% of patients treated with T-DM1 experienced elevated serum liver enzymes, a sign of hepatocellular injury.^4^ Managing this T-DM1-induced liver toxicity frequently necessitates interrupting treatment and modifying dosages.^2^

More recently, another AMC, mirvetuximab soravtansine-gynx (Elahere) received FDA approval for folate receptor α (FRα)-positive platinum-resistant ovarian cancer. In this AMC, the anti-FRα antibody is conjugated to another maytansine derivative, DM4, via a cleavable disulfide linker. In addition to these two FDA-approved AMCs, several more DM1- and DM4-based AMCs are currently in phase II/III clinical trials.^3^

Engineered scaffold proteins (ESPs) have become a promising alternative to mAbs for tumor targeting. These nonimmunoglobulin-binding proteins are substantially smaller than full-length mAbs, offering advantages such as enhanced extravasation and improved tissue penetration, while they can be engineered for high-affinity binding to cancer-related targets.^5,6^

Affibody molecules represent a particularly promising class of ESPs. Derived from the B-domain of staphylococcal protein A, these small proteins (58 amino acids, 6–7 kDa) have emerged as effective targeting agents due to their engineered high binding affinity, specificity, and favorable pharmacokinetic properties.^5^

Affibody-drug conjugates (AffiDCs) have been explored as promising alternatives or complements to ADCs. In particular, the Z_HER2:2891_ anti-HER2 affibody, with its high binding affinity (*K*_D_ = 76 pM), has proven effective as a carrier for the cytotoxic DM1 molecule. In most studies, this affibody is fused with an albumin binding domain (ABD) to extend its circulation half-life.^7–11^

A preclinical therapy study with mice bearing HER2-expressing SKOV3 xenografts has demonstrated the efficacy of this strategy. Four once-weekly injections of Z_HER2:2891_-ABD-E_3_-mcDM1 led to efficient tumor regression in all treated animals, with complete regression observed in some cases. The study evaluated two dosage groups: 15.1 mg/kg and 10.3 mg/kg. At the higher dose (15.1 mg/kg), weight loss was observed in two out of ten animals, suggesting this level approaches the maximum tolerated dose for this AffiDC. In contrast to ADCs which are primarily cleared by the liver, kidney excretion is the dominating route of elimination of Z_HER2:2891_-ABD-E_3_-mcDM1.^8^

While AffiDCs show promising results in preclinical models with effective tumor targeting, complementary strategies may further enhance the therapeutic potential of affibody-mediated delivery of cytotoxic drug payloads. To further improve the therapeutic window and reduce the systemic exposure, we have explored an alternative approach that temporally separates the targeting and therapeutic components, namely pretargeting.

Originally developed for radiotherapy applications, pretargeting typically involves sequential administration: first, a nonradiolabeled targeting molecule binds to the tumor and clears from circulation, followed by a small effector molecule carrying the therapeutic payload that rapidly binds to the prelocalized targeting agent while quickly clearing from nontarget tissues. Various pretargeting systems have been explored, including bispecific antibodies with hapten recognition, streptavidin–biotin interactions, bioorthogonal click chemistry, complementary oligonucleotide pairs and, more recently, supramolecular host–guest complexation.^12^

We have previously developed pretargeting strategies for radionuclide therapy based on highly selective peptide nucleic acid (PNA) duplex formation in vivo. When combined with Z_HER2:342_-mediated targeting of HER2-expressing tumors, this approach significantly improved tumor-to-kidney absorbed dose ratios compared to direct targeting across several studies.^13–16^ In a preclinical study, affibody-mediated pretargeting with a ^177^Lu-labeled secondary probe, [^177^Lu]­Lu-HP2, significantly extended median survival of treated tumor-bearing mice (66 vs 32 days) compared to the secondary probe alone.^17^ Combining PNA-based pretargeting with trastuzumab further improved survival over monotherapies, with no observed side effects or toxicity.^18^ Affibody molecules are particularly well-suited for pretargeting applications due to their slow internalization kinetics after binding to tumor cells. For example, in SKOV-3 cells, only 10 ± 1% of ^177^Lu-labeled Z_HER2:342_-HP9 primary agent had internalized after 24 h of incubation, while maintaining high cell-surface availability,^16^ a key prerequisite for effective binding of secondary effector molecules. However, slow internalization may limit the efficiency of pretargeting strategies in cases where the secondary probe carries a cytotoxic payload such as DM1, which requires internalization and lysosomal processing to exert its effect.

In this study, we have explored the potential of combining drug conjugates with our PNA-based pretargeting system. By replacing the therapeutic radionuclide of the secondary probe with DM1, and modifying certain residues to support this transition, we explore the characteristics of the secondary probes for future therapy applications. This novel DM1 delivery approach combines the potential benefits of pretargeting, such as an improved therapeutic window and reduced off-target effects, with the potent cytotoxicity of a maytansinoid.

We selected Z_HER2:2891_, a stabilized, more hydrophilic variant of the Z_HER2:342_ affibody, as the tumor-targeting component, in combination with the 9-mer primary hybridization probe HP9. In an earlier study, the Z_HER2:342_-HP9:HP20 probe pair demonstrated high binding affinity (*K*_D_ = 550 pM). Z_HER2:342_-HP9:[^177^Lu]­Lu-HP20 combination effectively delivered radiation to tumors while significantly reducing the kidney uptake that typically limits radiotherapy dosing when using Affibody-based targeting approaches.^16^

The biophysical properties of the Z_HER2:2891_-HP9 primary agent were first characterized, confirming its high thermal stability and preserved affinity for the HER2 receptor. Given that the attachment of highly hydrophobic payloads has been shown to cause problems such as aggregation and accelerated clearance,^19^ we designed and synthesized six DM1-conjugated 8-mer PNA effector probes featuring strategic hydrophilic modifications to identify a secondary probe suitable for future in vivo pretargeting experiments. Each probe featured a cysteine residue for conjugation of DM1 via the cathepsin-cleavable maleimide-valine-citrulline-*p*-aminobenzylcarbamate (Mal-VC-PAB) linker, along with varying numbers of glutamate residues and/or an eight–unit polyethylene glycol linker (PEG_8_) spacer to enhance negative charge and hydrophilicity of the molecules. All constructs were equipped with a versatile chelator, 1,4,7,10-tetraazacyclododecane-1,4,7,10-tetraacetic acid (DOTA) for radiolabeling with radiometals and evaluation in biodistribution studies. Biodistribution profiles of the ^177^Lu-labeled DM1-conjugated secondary probes were evaluated in NMRI mice and compared to the non-DM1 control probe, [^177^Lu]­Lu-HP20, and retention and internalization in SKOV-3 cells were investigated for three of the most promising probe combinations, Z_HER2_-HP9:SP2/SP5/SPc

## Results and Discussion

### Design of DM1-Conjugated Secondary Probes

A series of DM1-conjugated secondary probes with systematic structural variations were designed and synthesized to investigate their biodistribution properties while maintaining efficient hybridization with the primary agent. As shown in Figure 1A and Table 1, six different variants (SP1–SP5 and SPc) were created, all containing the same 8-mer PNA sequence (acaccagg), derived from HP20, that has previously demonstrated high-affinity binding (*K*_D_ = 550 pM) to the complementary HP9 sequence conjugated to the primary targeting agent.^16^

**Figure 1:**
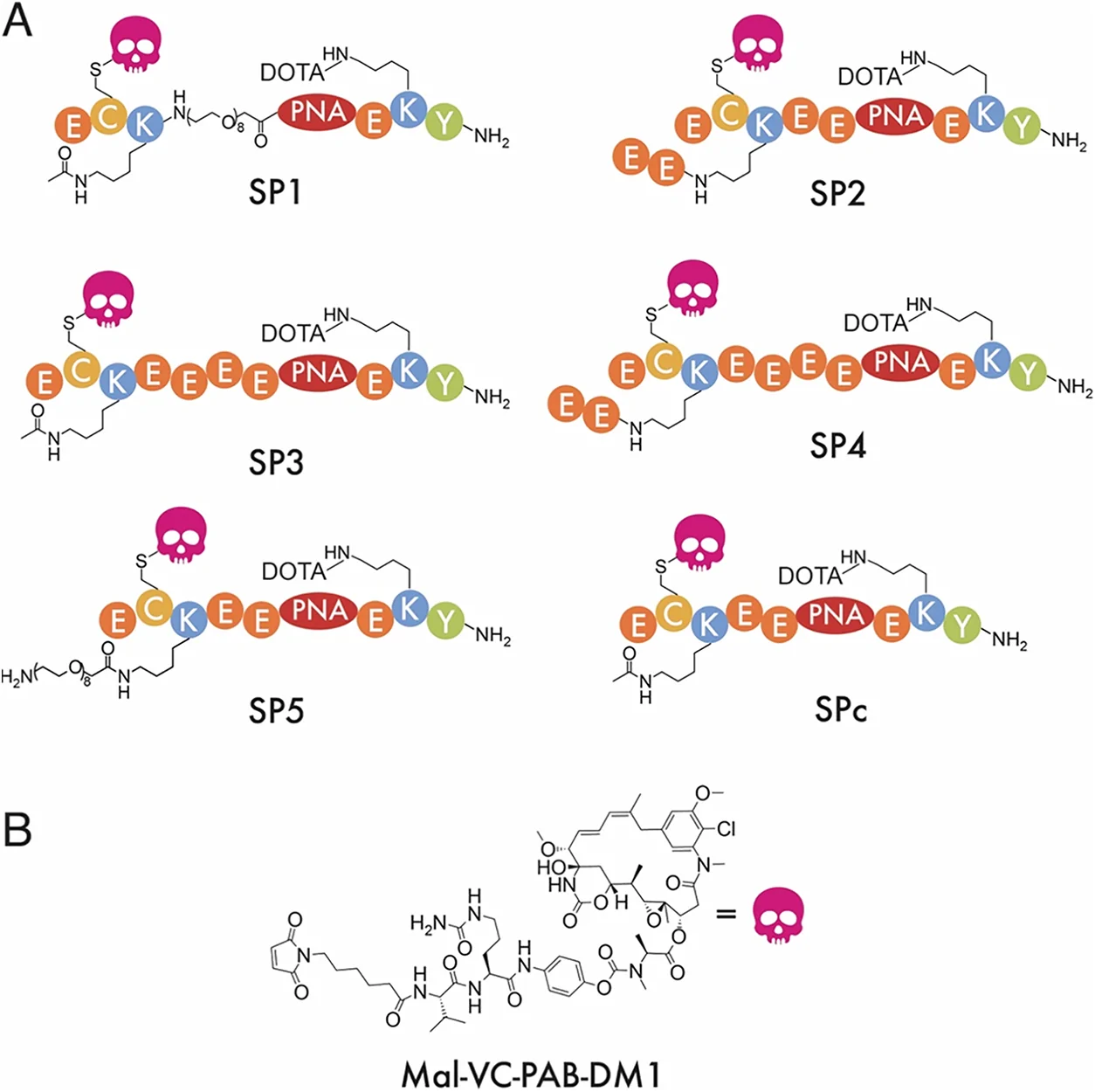
Design of DM1-conjugated secondary PNA probes. (A) Schematic representation of the six secondary probes (SP1–SP5 and SPc) featuring systematic structural variations. All constructs share core components: an 8-mer PNA hybridization domain (acaccagg; red oval), a cysteine residue (C, yellow circle) for DM1 conjugation, a DOTA chelator for radiolabeling, and a C-terminal tyrosine (Y, green circle). Variations between probes lie in the number and positioning of glutamate residues (E, orange circles), the presence and placement of PEG_8_ linkers, and overall molecular architecture. SP1 contains two glutamates and a PEG_8_ linker in a linear format. SP2 features a branched design with four glutamates on the backbone and two additional glutamates on a lysine side chain. SP3 has six glutamates in a linear configuration. SP4 combines six backbone glutamates with two branched glutamates on a lysine. SP5 incorporates four backbone glutamates and a PEG_8_ on the lysine side chain. SPc is the smallest variant, with four glutamates in a linear configuration. Lysine residues (K, blue circles) not used for functionalization were acetylated to neutralize positive charge. (B) Chemical structure of the cathepsin-cleavable Mal-VC-PAB-DM1 linker-payload complex, represented by the purple skull in panel **A**. The maleimide group enables site-specific conjugation to the cysteine residue in each probe.

**Table 1:** Sequences and Molecular Weights of the Primary Agent and the PNA-Based Secondary Probes Used in This Study^a^,^b^

name	sequence	theoretical mass (Da)	measured mass (Da)
Z_HER2:2891_-HP9	VDNKYAKEMRNAYWEIALLPNLTNQQKRAFIRKLYDDPSQSSELLSEAKKLNDSQAPKKLGSGSGSLPETGGGSS-cctggtgt-EK(DOTA)-PEG_2_-E-NH_2_	11,589	11,583
SP1	EC(DM1)-K(COCH_3_)-PEG_8_-EE-acaccagg-EK(DOTA)-Y-NH_2_	5306	5300
SP2	EC(DM1)-K(EE)-EE-acaccagg-EK(DOTA)-Y-NH_2_	5112	5113
SP3	EC(DM1)-K(COCH_3_)-EEEE-acaccagg-EK(DOTA)-Y-NH_2_	5155	5158
SP4	EC(DM1)-K(EE)-EEEE-acaccagg-EK(DOTA)-Y-NH_2_	5370	5366
SP5	EC(DM1)-K(PEG_8_)-EE-acaccagg-EK(DOTA)-Y-NH_2_	5263	5263
SPc	EC(DM1)-K(COCH_3_)-EE-acaccagg-EK(DOTA)-Y-NH_2_	4896	4895
HP20^b^	DOTA-PEG_2_-SS-acaccagg-EEY-NH_2_	3307	3310

a In the table, amino acids are represented in uppercase letters, while PNA monomers are denoted in lowercase letters. DM1 = Mal-VC-PAB-DM1, K­(COCH_3_) = lysine with an acetylated side-chain, DOTA = 1,4,7,10-tetraacacyclododecane-1,4,7,10-tetraacetic acid, PEG_2_ = (2-(2-aminoethoxy)­ethoxy)­acetic acid, PEG_8_ = 27-Amino-4,7,10,13,16,19,22,25-octaoxaheptacosanoic acid. The table also includes information about the theoretical mass and the experimental mass, as determined by MALDI-TOF.

b Affinity measurements determined by Westerlund et al.^14^

Each probe incorporated several key elements as detailed in Figure 1A and Table 1: (1) a cysteine residue for site-specific attachment of the cathepsin-cleavable Mal-VC-PAB-DM1 linker-payload complex (Figure 1B), (2) hydrophilic modifications including varying numbers of glutamate residues and/or a PEG_8_ linker to modulate charge and hydrophilicity, and (3) a DOTA chelator for radiolabeling to enable biodistribution studies.

To address the inherent hydrophobicity of the DM1 payload, we implemented different strategies across the probe series. SP1 and SP5 incorporated PEG_8_ linkers, in the backbone and in a branched configuration on a lysine side-chain, respectively, (Figure 1A and Table 1) to potentially shield the hydrophobic DM1 moiety, inspired by similar approaches in ADC design.^19–21^ In addition to PEG_8_, SP1 and SP5 carry two, respectively four, glutamates in the peptide backbone. SP2, SP3, and SP4 featured increasing numbers of glutamate residues (six–eight residues) positioned differently relative to the DM1 payload to investigate how charge distribution affects biodistribution. SPc is the smallest of the DM1-conjugated secondary probes and was designed with a linear arrangement with four glutamates within the peptide backbone.

We deliberately avoided using positively charged residues based on previous findings with anti-HER2 affibody molecules that showed increased hepatic uptake with positive charges.^22^ Instead, glutamate residues were used to create a negative charge profile, and any lysine residues not used for specific conjugations were acetylated to neutralize their positive charge. Previous studies have also demonstrated that incorporating glutamate residues near the DM1 payload increases hydrophilicity and reduces liver uptake of affibody-drug conjugates with albumin binding domains (AffiDC-ABD fusions).^8^

### Production and Analytical Verification of Primary and Secondary PNA-Probes

All DM1-containing secondary PNA-based probes were successfully synthesized using a combination of automated microwave-assisted solid-phase synthesis using previously optimized protocols^14^ and solution-phase drug conjugation, where maleimide–cysteine chemistry was employed to attach the cytotoxic payload to a unique cysteine residue.

The primary recognition tag, HP9, was synthesized as previously described^16^ and subsequently conjugated to the affibody Z_HER2:2891_-SR-H_6_ using Sortase A7+;^23^ a calcium-independent variant of the enzyme Sortase A.

Mass spectrometry analysis confirmed the successful synthesis of all primary and secondary probes with ≤ 6 Da agreement between theoretical and observed masses (Table 1).

The purity of the DM1-conjugated secondary probes was confirmed by RP-HPLC using an Agilent Zorbax SB-C18 column, with all probes showing ≥ 95% purity by peak integration at 220 nm (Figure S6).

### Biophysical and Binding Properties of Z_HER2_-HP9 and Secondary Probe Hybridization

Z_HER2:2891_-HP9 demonstrated high thermal stability with a melting temperature of 76 °C (Figure S1) and retained high binding affinity for the HER2 receptor with a K_D_ of 80 pM in SPR, similar to the previously investigated Z_HER2:342_-HP9 primary probe (K_D_ of 280 pM), confirming that PNA conjugation did not compromise the biophysical properties of the affibody and that the two variants of the HER2-binding primary probes have very similar binding kinetics (Figure S2, and Table 2). All measured DM1-conjugated secondary probes retained high-affinity hybridization to immobilized Z_HER2:2891_-HP9 or Z_HER2:342_-HP9 in SPR, with equilibrium dissociation constants ranging from 310 to 660 pM (Figure S3, and Table 2), indicating that the structural modifications did not significantly affect PNA duplex formation. A more detailed discussion on the binding properties of the secondary probes can be found in the Supporting Information.

**Table 2:** Kinetic Parameters Obtained from Fitting the Sensorgrams in Figures S2–S3 to a 1:1 Binding Model in the Biacore T200 Software

analyte	ligand	*k*_a_ (M^–1^ s^–1^)	*k*_d_ (s^–1^)	*K*_D_ (pM)	χ^2^
Z_HER2:2891_-HP9	HER2-Fc	4.5 × 10^6^	3.6 × 10^–4^	80	0.3
SP2	Z_HER2:2891_-HP9	1.4 × 10^5^	7.4 × 10^–5^	540	2.7
SP3	Z_HER2:2891_-HP9	1.5 × 10^5^	7.1 × 10^–5^	490	2.8
SP4	Z_HER2:2891_-HP9	1.1 × 10^5^	7.4 × 10^–5^	660	1.5
SP5	Z_HER2:2891_-HP9	1.3 × 10^5^	6.2 × 10^–5^	480	1.8
SP_C_	Z_HER2:2891_-HP9	2.2 × 10^5^	6.9 × 10^–5^	310	4.8
HP20	Z_HER2:2891_-HP9	2.1 × 10^5^	8.9 × 10^–5^	430	0.9

### Radiolabeling and In Vitro Stability of Secondary Probes

The DM1-conjugated secondary probes (SP1–SP5 and SPc), and the previously studied non-DM1 conjugated 8-mer HP20, were radiolabeled with 177Lu (Table 3). Most probes could be successfully labeled, with radiochemical yields (RCYs) ranging from 11% to 97%. The labeled probes demonstrated good stability when challenged with a 500-fold excess of EDTA. All labeled probes achieved 100% radiochemical purity (RCP) after purification using NAP-5 column. SP4 could not be effectively radiolabeled and was therefore excluded from further in vivo evaluations. The exact reason for this failure remains unclear, but it is possible that features such as the large clusters of glutamic acid residues induced unfavorable conformational changes, which limited the DOTA accessibility in SP4.

**Table 3:** Radiolabeling of the Probes with Lu-177^a^

secondary probe	radiochemical yield, % (*n* = 2)	radiochemical yield after 500-fold molar excess of EDTA, %	radiochemical purity, %
SP1	97 ± 2	85	100 ± 0
SP2	41 ± 3	37	100 ± 0
SP3	23 ± 1	28	100 ± 0
SP4	N.R		
SP5	22 ± 6	27	100 ± 0
SPc	11 ± 0	11	100 ± 0
HP20	53 ± 8	53	100 ± 0

a (N.R: No Reaction).

In vitro stability assay demonstrated high stability, with less than 10% release of free ^177^Lu from the radioconjugates up to 24 h of incubation in human serum. However, [^177^Lu]­Lu-SPc showed lower stability compared to the other variants (Figure S5).

### In Vivo Biodistribution of Secondary Probes in NMRI Mice

The success of pretargeting with secondary probes containing DM1 is dependent on a favorable biodistribution in vivo, ensuring no accumulation of toxin in any healthy, sensitive tissues. Therefore, DM1-coupled secondary probes were ^177^Lu-labeled and evaluated in nontumor-bearing NMRI mice at 4 h postinjection (Figures 2–3 and Table S1). All DM1-conjugated variants showed relatively rapid blood clearance, with blood levels ranging from 0.07 ± 0.01%ID/g for SP2 to 0.2 ± 0.1%ID/g for SPc. These values were significantly higher than those of the non-DM1 control probe HP20, which showed only 0.010 ± 0.003%ID/g, suggesting that conjugation of the DM1 payload prolongs blood circulation.

**Figure 2:**
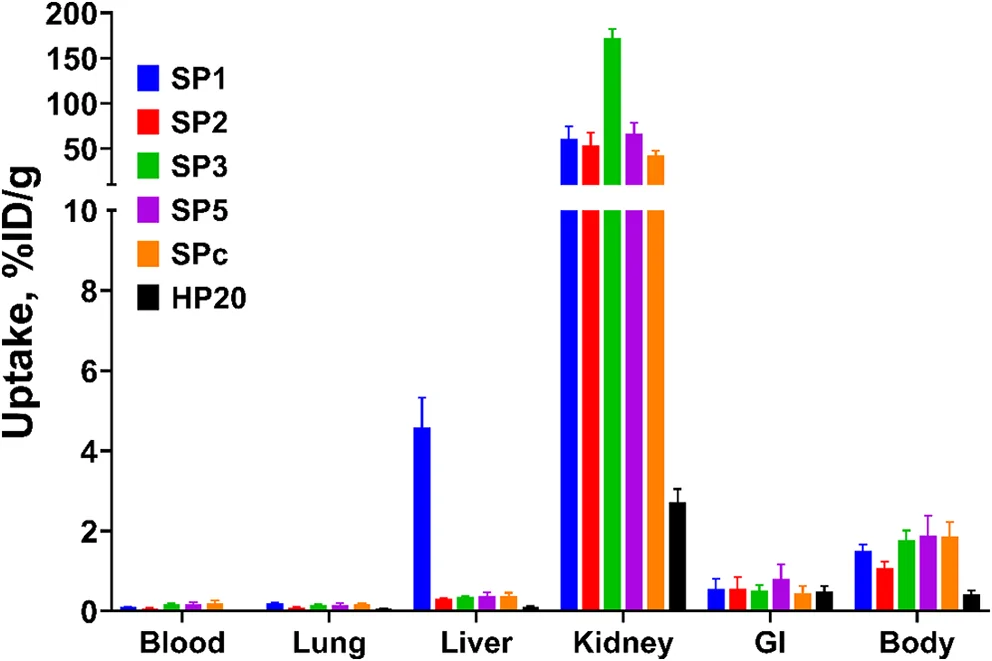
Biodistribution of DM1-PNA probes labeled with ^177^Lu in NMRI mice 4 h after injection. Data are presented as the average value from 4 samples ± SD.

**Figure 3:**
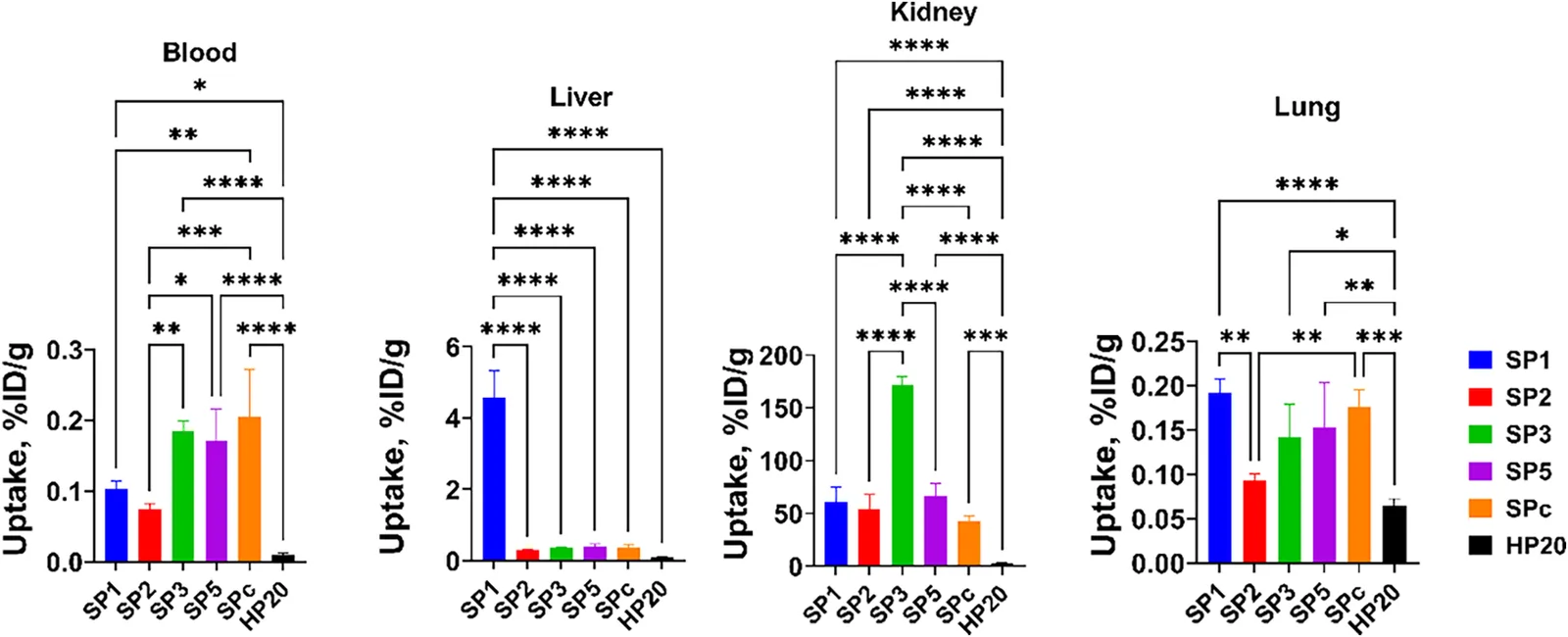
Biodistribution of DM1-PNA probes labeled with 177Lu in NMRI mice 4 h after injection. Data are presented as the average value from 4 samples ± SD. Asterisks mark a significant difference (*p* < 0.05). One-way ANOVA with Bonferroni’s multiple comparisons test was performed to find significant differences.

Despite consisting of similar building blocks, the probes displayed markedly different organ distribution profiles, particularly in the liver and kidneys. SP1 showed significantly (*p* < 0.05) higher liver accumulation (5 ± 1%ID/g) than any other probes (0.3–0.4%ID/g), a statistically significant difference (*p* < 0.05). For radiolabeled peptides, it is known that increased lipophilicity can lead to higher liver uptake.^24^ Reversed-phase high-performance liquid chromatography (RP-HPLC) analysis shows that SP1 has the longest retention time of the tested PNA probes (see Table S2), supporting the hypothesis that the high liver uptake is driven by lipophilicity. In contrast, SP3 exhibited the highest renal uptake (172 ± 10%ID/g), approximately three to four times higher than that of the other DM1-conjugated variants (43–67%ID/g). This outlier behavior cannot be explained by hydrophilicity, as the SP3 probe shows an intermediate retention time in the RP-HPLC analysis. Renal uptake proceeds through glomerular filtration followed by reabsorption in the proximal tubule, involving several possible transport mechanisms, which are not only affected by size and charge, but also by specific interactions with receptors.^25^ It has been shown for peptides that structural changes affect the uptake, as demonstrated by the exchange of every other l-glutamate with d-glutamate in a ^67^Ga-labeled (Glu)_14_ peptide, which significantly lowered the kidney uptake of an oligo-glutamic acid-containing peptide.^26^ In the present study, it is not obvious what modifications of the SP3 probe cause the observed high kidney uptake. Nevertheless, in a pretargeting setting, the accumulation of any cytotoxic agent in healthy organs is highly undesirable, and therefore, based on the unfavorable biodistribution of SP1 and SP3, they were both excluded from further evaluation.

SP2, SP5, and SPc demonstrated the most favorable biodistribution profiles, with relatively low liver uptake (0.3–0.4%ID/g) and moderate kidney accumulation (43.01–66.63%ID/g), without statistically significant differences. While SP5, like SP1, contains a PEG_8_ unit, it is arranged in a branched configuration on a lysine side chain and includes two additional glutamates. SP2 also carries a total of six glutamates, like SP3, but arranged in a branched configuration with two glutamates located on a lysine side chain near the DM1 payload. SPc, the smallest of the DM1-conjugated probes, shares the same core structure as SP2 and SP5 but has a simple lysine acetylation instead of additional modifications. These subtle differences in design appear to contribute significantly to their more favorable clearance profiles.

Although SP2, SP5 and SPc show favorable clearance patterns and good potential as secondary probes for pretargeting, their tumor cell-killing capabilities remain unexplored. Future evaluation in tumor-bearing mice is warranted to better understand how their structural variations affect tumor uptake. Notably, previous AffiDC therapy studies in SKOV3 xenograft models have shown that a kidney uptake of 81 ± 12%ID/g for ^99m^Tc-labeled Z_HER2:2891_-ABD-E_3_-mcDM1 was not associated with renal toxicity,^8^ supporting the safety of advancing these constructs. Interestingly, the non-DM1 HP20 probe exhibited markedly lower kidney accumulation (2.7 ± 0.3%ID/g). Similarly, the small molecule drug DOTA-TATE, with a primary clearance route via the kidneys, showed an increase in kidney retention when coupled to DM1,^27^ highlighting the considerable impact of DM1 conjugation on renal uptake. HP20 also trended toward lower hepatic accumulation than the DM1-conjugated variants, though this difference reached statistical significance only in comparison to SP1.

### Cell Binding and Internalization

The binding kinetics of the ^177^Lu-labeled secondary probe (SP2) pretreated with the primary Z_HER2:2891_-HP9 agent exhibited two affinity values, with the dominant one at *K*_D1_ = 31 ± 1 pM (64% weight) and the second affinity at *K*_D2_ = 6 ± 1 nM (36% weight) (Figure 4).

**Figure 4:**
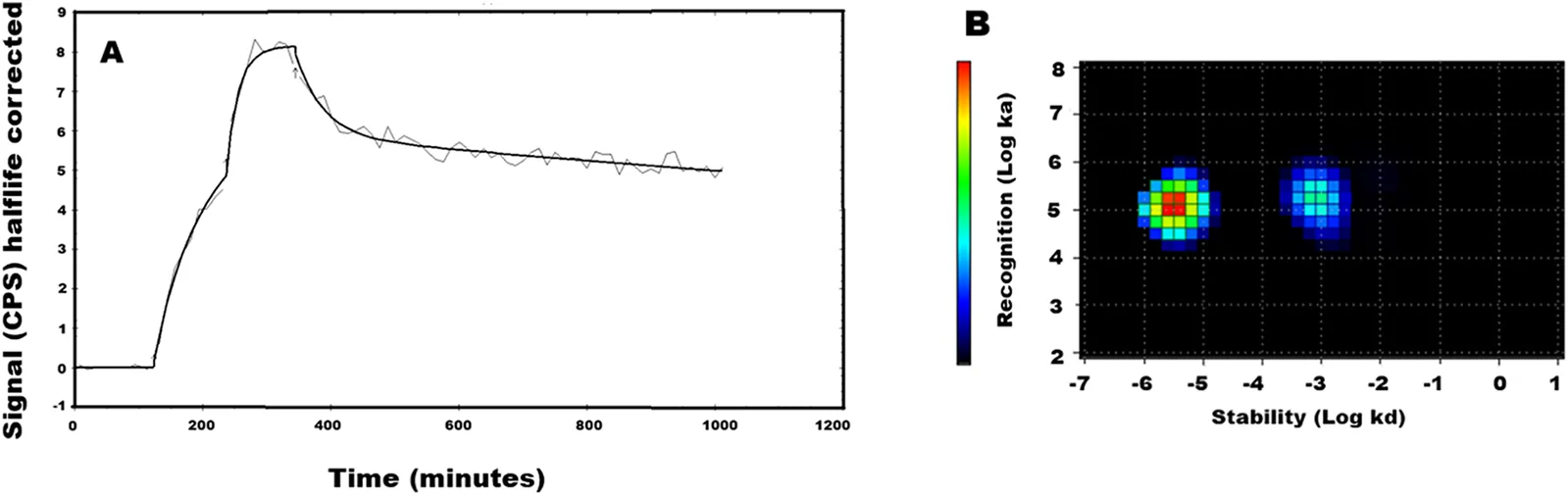
(A) Sensorgram obtained using LigandTracer and (B) Graphic presentation of InteractionMap analysis of interaction of [^177^Lu]­Lu-SP2 pretreated with Z-HP9 on SKOV-3 cells. The experiment was performed in duplicate.

Figure 5 displays the cellular processing and retention data for [^177^Lu]­Lu-SP2, [^177^Lu]­Lu-SP5, and [^177^Lu]­Lu-SPc preincubated with Z-HP9 on SKOV-3 cells. Both SP2 and SP5 showed an initial decrease in total cell-associated activity within the first hour, after which both probes stabilized. SP2 preincubated with to Z-HP9 maintained a total cell-associated activity of 26–32% from 4 h onward, with an internalized fraction of 12.1 ± 1.8% at 24h. SP5 pretreated with Z-HP9 stabilized at a higher level of 40–47% of total cell-associated activity, with an internalized fraction increasing progressively to 20.4 ± 2.5% at 24 h. The internalized fraction observed for Z-HP9:[^177^Lu]­Lu-SP5 was notably higher than previously reported for either the radiolabeled primary agent alone, [^177^Lu]­Lu-Z-HP9, or the non-DM1 complex Z-HP9:[^177^Lu]­Lu-HP20, both of which showed an internalized fraction of approximately 10% after 24 h,^16^ representing an approximately 2-fold increase. Z-HP9:[^177^Lu]­Lu-SPc displayed an atypical and highly variable cellular processing profile with an internalized fraction of 14.9 ± 0.62 at 24 h, and should be interpreted with caution. In contrast, the previously studied AffiDC [^99m^Tc]­Tc-Z_HER2:2891_-Mc-DM1, which uses the same affibody scaffold as in the present work, displayed a substantially faster internalization, with approximately 30% internalized in SKOV-3 cells already after 4 h of incubation.^7^ The slow internalization of Z-HP9 makes it an effective primary agent for pretargeting by ensuring prolonged availability of the primary probe on the cell surface for secondary probe hybridization. However, this same property may also limit the efficiency of intracellular delivery of a DM1-labeled secondary probe, as DM1 relies on internalization and lysosomal processing to exert its cytotoxic effect.

**Figure 5:**
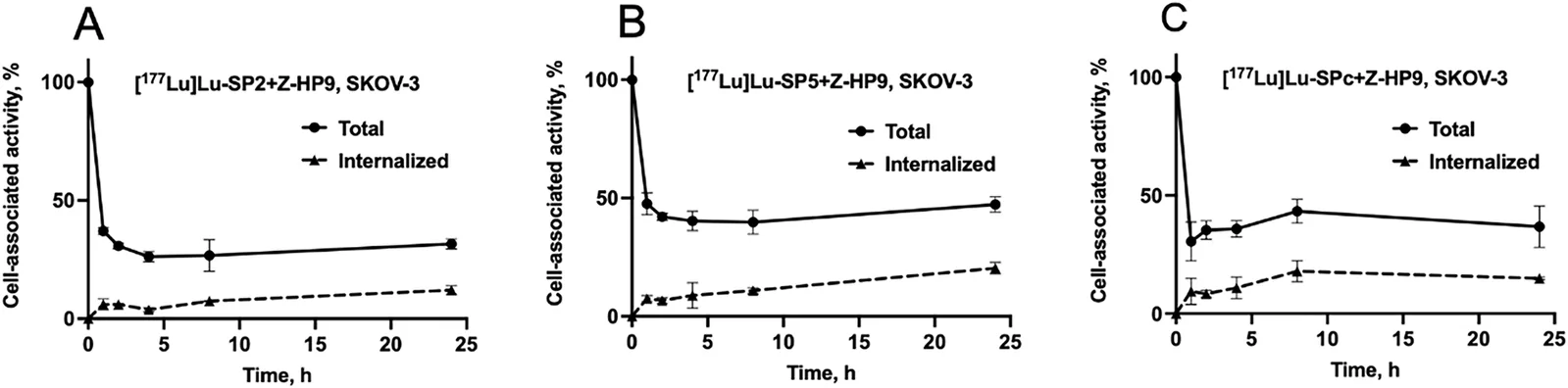
Retention and internalization of (A) [^177^Lu]­Lu-SP2, (B) [^177^Lu]­Lu-SP5, and (C) [^177^Lu]­Lu-SPc (10 nM) pretreated with Z-HP9 (1 nM, 1h) on SKOV-3 cells after interrupted incubation. The data are presented as the mean ± SD from three samples.

The optimal time frame for internalization is hard to determine. Previous studies examining cytotoxic pretargeting mediated by click-chemistry toward PSMA-positive cells revealed a significant difference in cell-killing when comparing the anti-PSMA antibody 5D3 with the faster internalizing Fab fragment of the same antibody.^28^ Cell-killing was more effective when cells were pretargeted with 5D3 compared to the Fab, showcasing the need to have efficient secondary probe binding at the cell surface before endocytosis. Ideally, the rate of internalization should initially be slow, but increase upon the secondary probe binding to the primary probe on cells, and a probe design to support such an effect would be of interest. It has been noted that the internalization efficiency drops from approximately 30% after 6 h for the dimeric formatted affibody (Z_HER2:2891_)_2_-ABD-Mc-DM1 down to 20% when adding 3 or 6 consecutive glutamic acid residues to the molecule ((Z_HER2:2891_)_2_-ABD-E_3_-Mc-DM1 or (Z_HER2:2891_)_2_-ABD-E_6_-Mc-DM1).^29^ This suggests that by reducing the number of clustered glutamate residues on the secondary probe a higher degree of endocytosis might be achieved. Another consideration for efficient cytotoxicity is the mindful placement of PEG molecules. A more hydrophilic drug can be achieved by branching the PEG molecule away from the peptide chain in contrast to placement within the chain.^30^ This can offer better shielding of the hydrophobic payload and a more biophysically stable drug, less prone to aggregation. Taking these considerations into account, SP5 emerges as a promising candidate with a favorable biodistribution profile, effective payload shielding with a branched PEG_8_-molecule, and fewer clustered glutamic acid residues. Therefore, it serves as a promising candidate for future evaluation in tumor-bearing mice or as a basis for further secondary probe engineering.

### Concluding Remarks

This study aimed to design and biophysically characterize a set of novel PNA-based probes coupled to the cytotoxic agent DM1 to establish their feasibility as therapeutic secondary probes in a pretargeting setting. The primary targeting agent, Z_HER2:2891_-HP9, exhibited excellent thermal stability (*T*_m_ = 76 °C) and retained high affinity for HER2 (*K*_D_ = 80 pM), confirming its suitability for pretargeting applications.

We investigated six DM1-conjugated secondary probes, each featuring an identical 8-mer PNA sequence complementary to HP9 but differing in their number of glutamates, PEG_8_ incorporation, and overall architecture. All studied probes displayed comparable binding kinetics (*K*_D_ = 310–660 pM), indicating that modifications outside the hybridization domain do not compromise duplex formation in vitro.

Biodistribution measurements in normal, nontumor-bearing mice revealed notable differences in organ accumulation. SP1 showed elevated liver uptake (5%ID/g), while SP3 exhibited significantly higher kidney retention (172%ID/g). In contrast, SP2, SP5, and SPc demonstrated more favorable profiles, with lower hepatic (0.3–0.4%ID/g) and renal (43–67%ID/g) uptake. Cellular internalization studies of SP2 and SP5 prehybridized with Z_HER2:2891_-HP9 revealed differences in HER2-mediated endocytosis. SP2 reached a maximum internalization fraction of 12.1% after 24 h while SP5 reached 20.4%, potentially making SP5 more cytotoxic.

## Methods

### Synthesis and Purification of PNA-Based DM1-Conjugated Hybridization Probes

The synthesis of primary and secondary PNA probe backbones followed a standard Fmoc solid phase peptide synthesis (SPPS) strategy (see Supporting Information for a more detailed description).

Following the complete synthesis of secondary probe main chains, the Mmt group on probes synthesized with Lys­(Mmt)-(OH) (for attachment of side chain modifications in SP2, SP4 and SP5) was selectively deprotected. This was done by repeated resin washes with acetic acid (AcOH):trifluoroethanol (TFE):DCM:TIS (10:20:69:1) until the liquid turned yellow then reverted back to transparent. Following the Mmt deprotection the resin was neutralized by washing once with 1% DIEA in DMF. Residues coupled on the Lys side chain were then coupled using automated SPPS when coupling AA residues and manually when coupling PEG_8_, with identical coupling strategies as for main chain residues (see Supporting Information).

After peptides were fully synthesized, they were cleaved off the solid support by TFA-cleavage and purified by RP-HPLC as described in more detail in the Supporting Information

For conjugating DM1 to the secondary probes, purified secondary probe was initially dissolved in phosphate-buffered saline (PBS) to a concentration of 1–5 mg/mL. Tris­(2-carboxyethyl)­phosphine (TCEP) was then added to a final concentration of 5 mM, and the solution left on a rotamixer for 1 h at 37 °C. Subsequently equimolar amounts of Mal-VC-PAB-DM1 (MedChemExpress) were added and the mixture diluted in PBS to a final concentration of 100 μM for both the toxin and the PNA probe. The mixture was then placed in a rotamixer and left overnight at room temperature. The following day, the conjugation mixture was filtered through a 0.45 μm filter and purified by RP-HPLC at 45 °C with a gradient of 20–60% buffer B over 50 min. Peaks were analyzed by MS MALDI-TOF, and fractions containing the correct product were collected and pooled together before being freeze-dried overnight. Freeze-dried and pure DM1-conjugated secondary probes were resuspended in PBS and concentrations were measured by UV absorbance at 260 nm. Extinction coefficients of PNA probes were calculated by summing the total absorbance values of PNA bases at 260 nm present within the sequence of the probes (13,700 M^–1^cm^–1^ for a, 6600 M^–1^cm^–1^ for c, 11,700 M^–1^ cm^–1^ for g and 8600 M^–1^ cm^–1^ for t).

### Cell Binding and Internalization

To evaluate the binding affinity of the radiolabeled conjugate to HER2 receptors and the cell-bound primary probe, their binding and dissociation kinetics from SKOV-3 cells were measured using a LigandTracer white instrument (Ridgeview Instruments AB, Vänge, Sweden) as described previously.^31^ Briefly, cells were incubated with the primary agent, Z_HER2:342_-HP9 or Z_HER2:2891_-HP9, for 2 h (1 nM). The media was replaced with fresh media, and the secondary probe [^177^Lu]­Lu-SP2 was added to obtain concentrations of 1 and then 5 nM. Thereafter, the radioactive medium was replaced with fresh one, and the dissociation curve was recorded for several hours. The data were analyzed by InteractionMap software (Ridgeview Instruments AB, Uppsala, Sweden) to calculate the association and dissociation rates, and dissociation constant at equilibrium (K_D_).

Internalization and retention of radiolabeled probes in SKOV-3 cells were assessed using a validated acid-wash method.^32^ Briefly, cells were incubated with the primary agent (1 nM) for 1 h at 4 °C, followed by washing and incubation of the cells with radiolabeled secondary probes (10 nM) for 30 min at 4 °C. After washing, cells were incubated in complete medium at 37 °C for 1, 2, 4, 8, and 24 h. The radioactivity of acidic and alkaline fractions was considered as membrane and internalized parts, respectively. The experiment was done in triplicate.

### Radiolabeling of Probes with ^177^Lu and In Vitro Stability

For the radiolabeling with ^177^Lu, 70 μL of 0.2 M of NH_4_Ac, pH 5.5 was added to 30 μg of the probe (30 μL of 1 mg/mL stock in PBS). [^177^Lu]­LuCl_3_ was added and the labeling mixture was incubated at 95 °C for 1 h. RCY was then analyzed using instant thin-layer chromatography (iTLC) developed with 0.2 M citric acid, pH 2.0. To remove loosely bound ^177^Lu, a treatment with an excess of ethylenediaminetetraacetic acid tetra sodium salt (EDTANa_4_) was performed, and the reaction mixture was incubated at 95 °C for 10 min. The purification was performed using a NAP-5 size-exclusion column, pre-equilibrated, and eluted with 1% BSA in PBS. Table 3 shows the results of the labeling with ^177^Lu.The stability was assessed by incubating a fraction of purified radioconjugate (613 pmol) with human serum (100 μL) at 37 °C to mimic the concentration of the tracer in murine blood immediately after injection. At 1, 4, and 24 h postincubation, 1 μL of the aliquots was taken to evaluate stability using instant thin-layer chromatography (iTLC), with 0.2 M sodium citrate (pH 2.5) as the mobile phase. As a control, free ^177^Lu was incubated with human serum.

### Biodistribution Study of ^177^Lu-Labeled Probes

Animal experiments were performed according to the national legislation for work with laboratory animals and the permit granted by the Ethical Committee for Animal Research in Uppsala (permit 5.8.18–00473/2021, approved 26 February 2021). For biodistribution studies, female NMRI mice were randomized into groups of four. The average weight of the animal was 29.7 ± 2.9 g. The mice were intravenously injected with the probes (3 μg, 613 pmol in 100 μL PBS). Four hours after injection, all mice were euthanized, and the organs of interest were collected and weighed, and their radioactivity was measured. The percentage of the total injected dose per gram of sample (% ID/g) was calculated. For comparison, the biodistribution of [^177^Lu]­Lu-HP20 (2 μg, 613 pmol in 100 μL PBS) was measured in the same way. No unexpected or unusually high safety hazards were encountered in this study.

## Supplementary Material



## Data Availability

The generated data is available upon request from the corresponding authors.

## Notes

Additional experimental details regarding materials and methods. Stability analysis of Z-HP9 by CD spectroscopy. SPR sensorgrams of Z-HP9 binding to HER2. SPR sensorgrams of secondary probe binding to Z-HP9. Detailed biodistribution data of secondary probes in NMRI mice. In vitro stability and iTLC profiles of secondary probes. Purity and hydrophobicity analysis of secondary probes by analytical RP-HPLC (PDF)
