# Supplementary material for: Peptide Nucleic Acid Probes for Pretargeted Affibody-Mediated Delivery of Cytotoxic Payloads to HER2-Positive Carcinoma
Source: Bioconjug Chem. 2026 Jul 30;37(8):1562–70. doi: 10.1021/acs.bioconjchem.6c00035 (PMC13495548; doi:10.1021/acs.bioconjchem.6c00035)
Supplement: Supplementary file 1 [file bc6c00035_si_001.pdf]

Supporting Information:

Peptide nucleic acid probes for pretargeted affibody-mediated delivery of cytotoxic payloads to HER2-positive carcinoma

Jacob Clinton[1,ǂ], Kristina Westerlund[1,ǂ], Astrid Haraldsson[1], Ebba Nordén[1], Tilde Hagelin[2], Eleftherios Papalanis[2], Moeen Ud-din[2], Anzhelika Vorobyeva[2], Torbjörn Gräslund[1], Amelie Eriksson Karlström[1] and Maryam Oroujeni[2,*]

[1]Department of Protein Science, School of Engineering Sciences in Chemistry, Biotechnology and Health, KTH Royal Institute of Technology, Stockholm 10691, Sweden;
[2]Department of Immunology, Genetics and Pathology, Uppsala University, Uppsala 75185, Sweden

ǂ Equally contributed

*Correspondence: Maryam Oroujeni, +46-733 28 92 94

Biophysical Characterization of the Primary Agent $Z_{HER2:2891}$-HP9

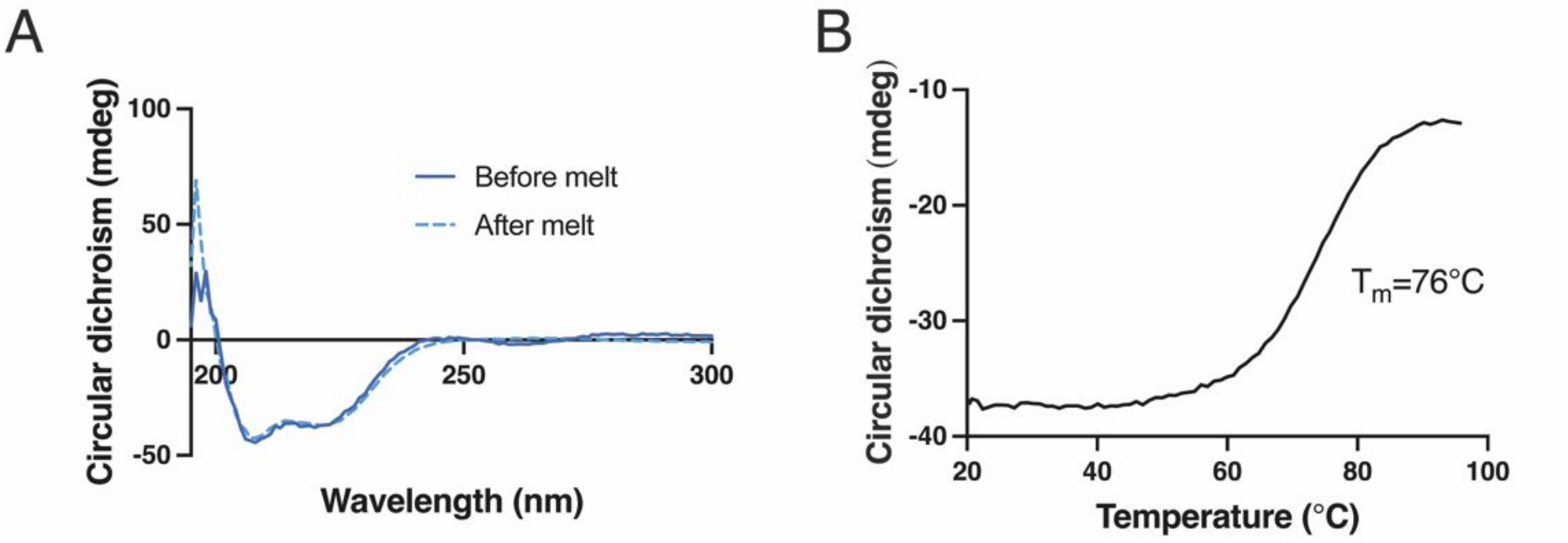

Figure S1. *CD spectra of $Z_{HER2:2891}$-HP9 before (solid lines) and after (dash lines) heat treatment at 100°C (**A**). The loss of secondary structure as a function of temperature was followed by monitoring the CD intensity at 221 nm for $Z_{HER2:2891}$-HP9 (**B**).*

For the pretargeting application, not only is the biodistribution of the secondary probes important, but their high-affinity interaction with the primary agent is critical. To study the interaction between the primary and secondary agents, the primary affibody-PNA conjugate $Z_{HER2:2891}$-HP9 was produced and characterized. The thermal stability of $Z_{HER2:2891}$-HP9 was evaluated using circular dichroism (CD) spectroscopy. As shown in Figure S1A, the CD spectra before and after heat treatment at 100 °C were nearly identical, exhibiting characteristic α-helical minima at 207 and 221 nm, indicative of a highly alpha-helical conjugate that could efficiently refold after denaturation. Thermal denaturation, monitored at 221 nm, revealed a melting temperature (T_m) of 76 °C (Figure S1B), consistent with the previously reported T_m of DOTA-conjugated $Z_{HER2:2891}$[1]. This demonstrates that PNA conjugation does not compromise affibody stability. Furthermore, $Z_{HER2:2891}$-HP9 exhibited a 10 °C higher T_m than our previously studied construct $Z_{HER2:342}$-HP9 (T_m = 66 °C;[2]), reflecting the enhanced thermal and chemical stability of the optimized $Z_{HER2:2891}$ scaffold, which retains the HER2 binding surface while offering improved hydrophilicity[3].

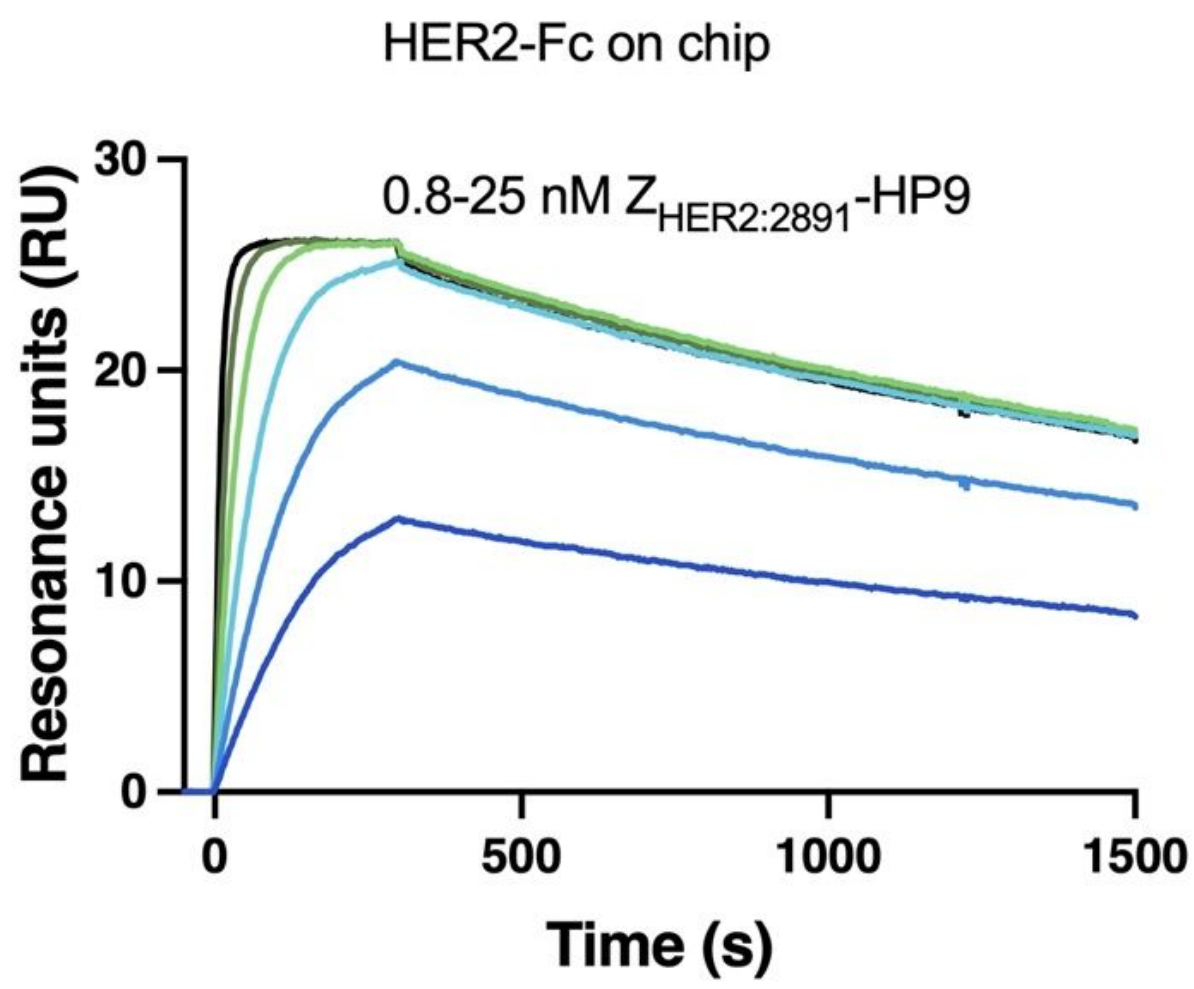

Figure S2. *Representative SPR sensorgram depicting the interaction between immobilized human HER2 receptor and $Z_{HER2:2891}$-HP9 at 25°C. $Z_{HER2:2891}$-HP9 was introduced at six different concentrations (0.8, 1.6, 3.1, 6.3, 12.5, and 25 nM) and was monitored during a 20-minute dissociation period after injection for 300 seconds. All measurements were conducted in duplicate and are presented after double referencing (subtracting data from the reference channel and buffer injection).*

To assess HER2 binding, SPR analysis was performed at 25 °C. As shown in Figure S2 and summarized in Table 2, $Z_{HER2:2891}$-HP9 displayed high-affinity binding to immobilized HER2, characterized by a rapid association rate and slow dissociation rate. The resulting equilibrium dissociation constant (K_D) was estimated to be 80 pM, closely matching the 76 pM previously reported for $Z_{HER2:2891}$ with a C-terminal DOTA[1]. Kinetic constants were also in good agreement with those determined for $Z_{HER2:342}$-HP9 ($k_a = 1.0 \times 10^6$ M^{-1} s^{-1}, $k_d = 2.9 \times 10^{-4}$ s^{-1}, K_D = 280 pM;[4]), confirming that HP9 conjugation does not impair HER2 binding.

Surface Plasmon Resonance Analysis of Secondary Probe Hybridization to Z-HP9

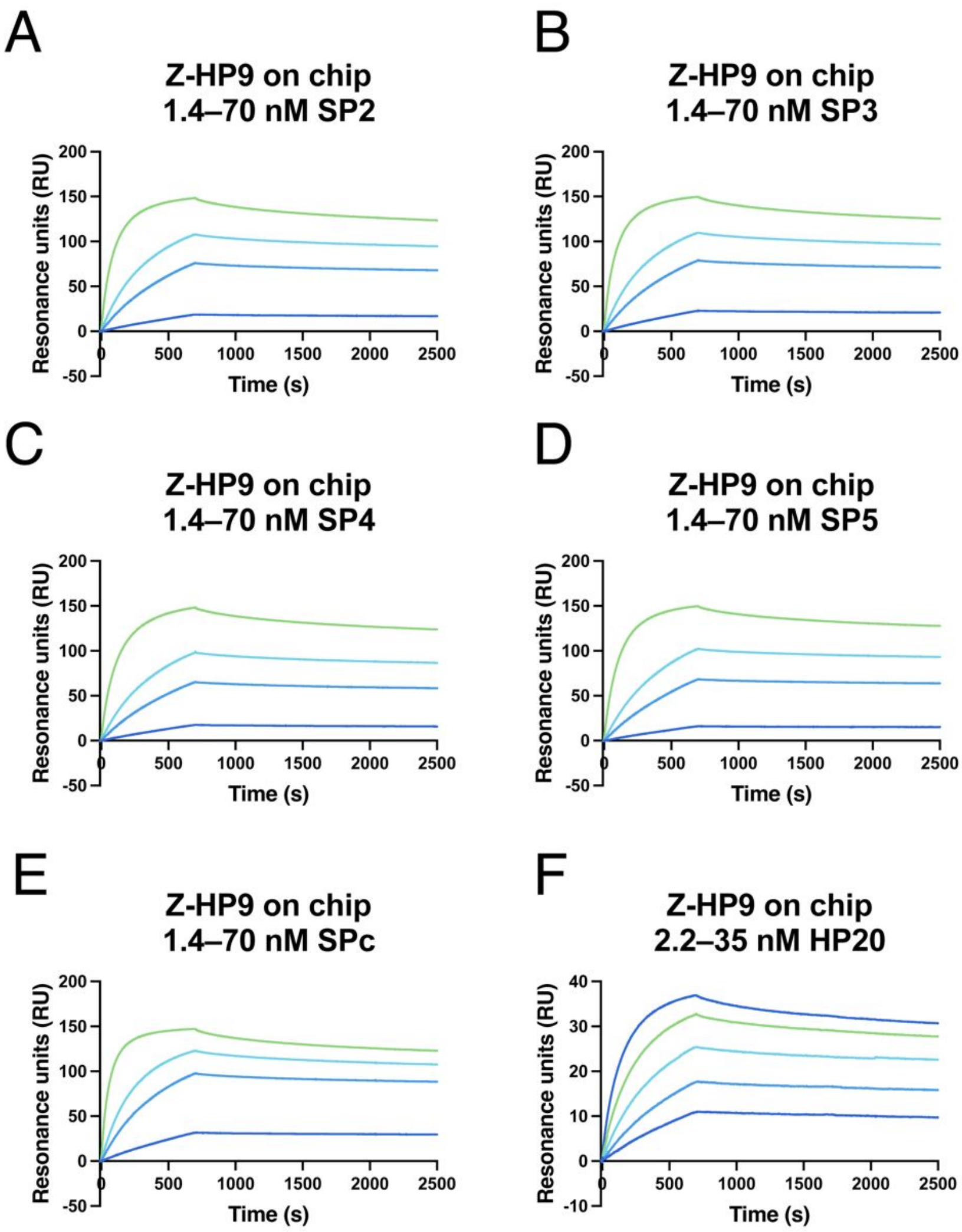

Figure S3. *Representative SPR sensorgrams depicting the interaction between the immobilized primary probe $Z_{HER2:2891}$-HP9 with the DM1-containing secondary probes SP2 (**A**), SP3 (**B**), SP4 (**C**), SP5 (**D**) and SP_C (**E**). The secondary probes were introduced at four different concentrations (1.4, 7, 14, and 70 nM at 25°C) and monitored during a 30-minute dissociation period after injection for 700 seconds. For comparison, HP20 was injected at concentrations of 2.2, 4.4, 8.8, 17.5, and 35 nM over another chip surface with immobilized $Z_{HER2:2891}$-HP9 in **F**. All experiments in (**A−F**) were done in duplicates, and the sensorgrams are double-referenced by subtraction of a reference surface and a buffer injection.*

The hybridization of the DM1-conjugated secondary PNA probes (SP2–SP5 and SPc) to immobilized ZHER2:2891-HP9 was evaluated by SPR at 25°C (Figure S3A-E). SP1 was excluded from further biophysical characterization due to its unfavorable liver uptake observed in vivo. For comparison, the hybridization of SP3 to $Z_{HER2:342}$-HP9 (Figure S3C) and of HP20, a non-DM1-conjugated 8-mer PNA probe with identical base sequence, to $Z_{HER2:2891}$-HP9 (Figure S3G) was also analyzed under similar conditions. Interestingly, all analyzed probes showed remarkably similar binding kinetics despite their structural differences. Association rates ranged from 1.1×10^5 to 2.2×10^5 M^{-1} s^{-1}, while dissociation rates were between 6.2×10^{-5}

and 8.9×10^{-5} s^{-1}. These kinetics translated to K_D values ranging from 310 to 660 pM. The binding affinity of the non-DM1 probe HP20 (K_D=430 pM) fell within this range, indicating that neither the conjugation of the bulky Mal-VC-PAB-DM1 moiety to the cysteine side chain nor the introduction of hydrophilic modifications (PEG_8 linker and glutamate residues) significantly affected the hybridization properties of the PNA sequence.

These values are in good agreement with the previously reported K_D of 550 pM (k_a=1.2×10^5 M^{-1} s^{-1}, k_d=6.6×10^{-5} s^{-1}) for the interaction between $Z_{HER2:342}$-HP9 and HP20 at 25°C;[2]. The consistent binding properties across all variants suggest that the PNA hybridization domain functions independently of the surrounding modifications, providing flexibility in probe design to optimize other properties.

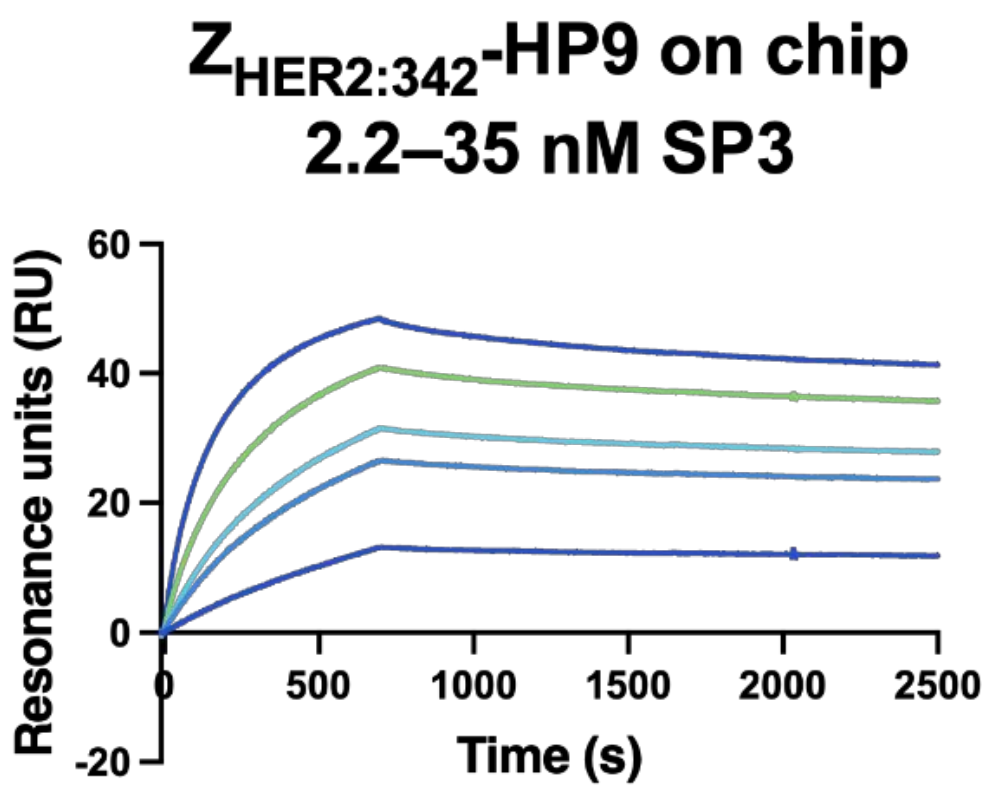

Figure S4: *Representative SPR sensorgrams depicting the interaction between the immobilized primary probe $Z_{HER2:342}$-HP9 with the DM1-containing secondary probes SP3. SP3 was injected at concentrations of 2.2, 4.4, 8.8, 17.5, and 35 nM over immobilized $Z_{HER2:342}$-HP9. The measurements were done in duplicates, and the sensorgrams are double-referenced by subtraction of a reference surface and a buffer injection.*

To confirm that switching from our previously studied HER2-targeting primary agent, $Z_{HER2:342}$-HP9, to $Z_{HER2:2891}$-HP9 did not affect the secondary probe's ability to bind the primary agent, we also examined the interaction between one of the DM1-containing probes, SP3, and $Z_{HER2:342}$-HP9 (Figure S4). SP3 bound $Z_{HER2:342}$-HP9 with a K_D of 340 pM (k_a = 2.0×10^5 $M^{-1}s^{-1}$, k_d = 6.8×10^{-5} s^{-1}) and $Z_{HER2:2891}$-HP9 with a comparable K_D of 490 pM, both in good agreement with the previously reported K_D of 550 pM (k_a = 1.2×10^5 $M^{-1}s^{-1}$, k_d = 6.6×10^{-5} s^{-1}) for the interaction between $Z_{HER2:342}$-HP9 and HP20 at 25°C;[2]. The consistent binding properties across all variants suggest that the PNA hybridization domain functions independently of the surrounding modifications, providing flexibility in probe design to optimize other properties.

Table S1. *Biodistribution of DM1-PNA probes labeled with Lu-177 in NMRI mice 4 h after injection.*

	Uptake, %ID/g					
Organ or Tissue	SP1	SP2	SP3	SP5	SPc	HP20
Blood	0.10±0.01[d,e]	0.07±0.01[f,g,h]	0.18±0.02[f,l]	0.17±0.05[g,n]	0.2±0.1[d,h,o]	0.010±0.003[e,l,n,o]
Lung	0.19±0.02[a,e]	0.09±0.01[a,h]	0.16±0.02[l]	0.15±0.05[n]	0.18±0.02[h,o]	0.06±0.01[e,l,n,o]
Liver	5±1[,b,c,d,e]	0.30±0.02[a]	0.35±0.03[b]	0.4±0.1[c]	0.4±0.1[d]	0.11±0.01[e]
Kidney	61±14[,e]	54±14[f,i]	172±10[b,f,j,k,l]	67±12[j,n]	43±5[k,o]	2.7±0.3[e,i,l,n,o]
GI*	0.6±0.3	0.6±0.3	0.5±0.2	0.8±0.4	0.5±0.2	0.5±0.1
Carcass*	1.5±0.2	1.1±0.2	1.8±0.2	1.9±0.5	1.9±0.45	0.4±0.1

[a] Significant ($p<0.05$) difference between [^{177}Lu]Lu-SP1 and [^{177}Lu]Lu-SP2
[b] Significant ($p<0.05$) difference between [^{177}Lu]Lu-SP1 and [^{177}Lu]Lu-SP3
[c] Significant ($p<0.05$) difference between [^{177}Lu]Lu-SP1 and [^{177}Lu]Lu-SP5
[d] Significant ($p<0.05$) difference between [^{177}Lu]Lu-SP1 and [^{177}Lu]Lu-SPc
[e] Significant ($p<0.05$) difference between [^{177}Lu]Lu-SP1 and [^{177}Lu]Lu-HP20
[f] Significant ($p<0.05$) difference between [^{177}Lu]Lu-SP2 and [^{177}Lu]Lu-SP3
[g] Significant ($p<0.05$) difference between [^{177}Lu]Lu-SP2 and [^{177}Lu]Lu-SP5
[h] Significant ($p<0.05$) difference between [^{177}Lu]Lu-SP2 and [^{177}Lu]Lu-SPc
[i] Significant ($p<0.05$) difference between [^{177}Lu]Lu-SP2 and [^{177}Lu]Lu-HP20
[j] Significant ($p<0.05$) difference between [^{177}Lu]Lu-SP3 and [^{177}Lu]Lu-SP5
[k] Significant ($p<0.05$) difference between [^{177}Lu]Lu-SP3 and [^{177}Lu]Lu-SPc
[l] Significant ($p<0.05$) difference between [^{177}Lu]Lu-SP3 and [^{177}Lu]Lu-HP20
[m] Significant ($p<0.05$) difference between [^{177}Lu]Lu-SP5 and [^{177}Lu]Lu-SPc
[n] Significant ($p<0.05$) difference between [^{177}Lu]Lu-SP5 and [^{177}Lu]Lu-HP20
[o] Significant ($p<0.05$) difference between [^{177}Lu]Lu-SPc and [^{177}Lu]Lu-HP20
* data for the GI tract, with content and carcass, are presented as % of injected dose per whole sample.

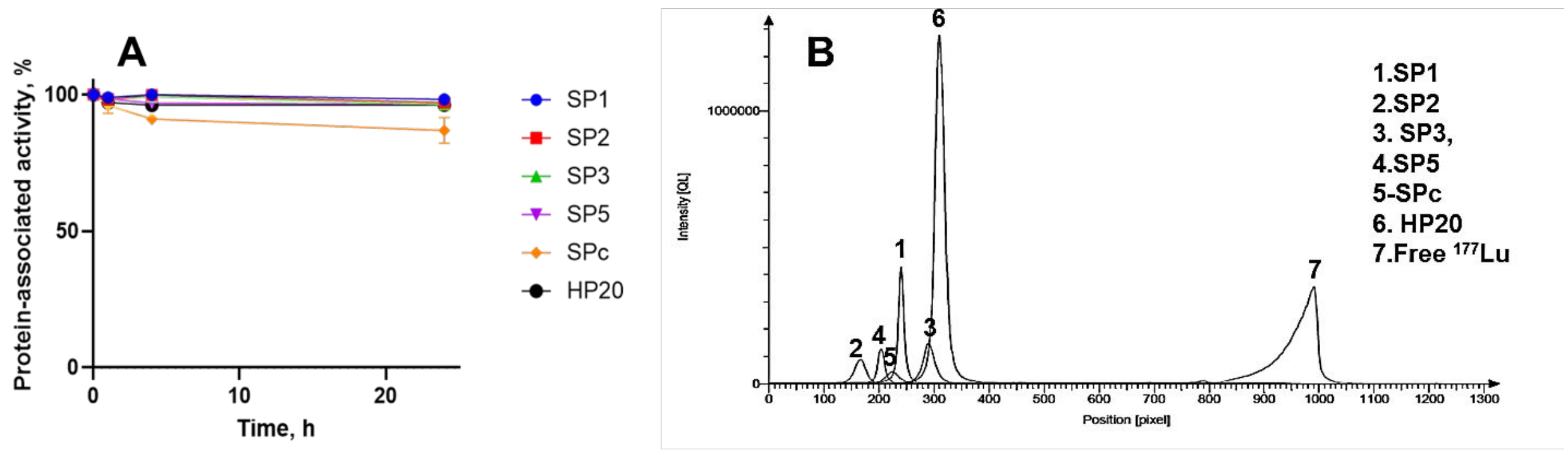

Figure S5. *A) In vitro stability, and **B)** iTLC profile of ^{177}Lu-labeled probes and free ^{177}Lu incubated in human serum at 37 ℃. The experiment was performed in duplicate. iTLC profile was provided for 4 h incubation.*

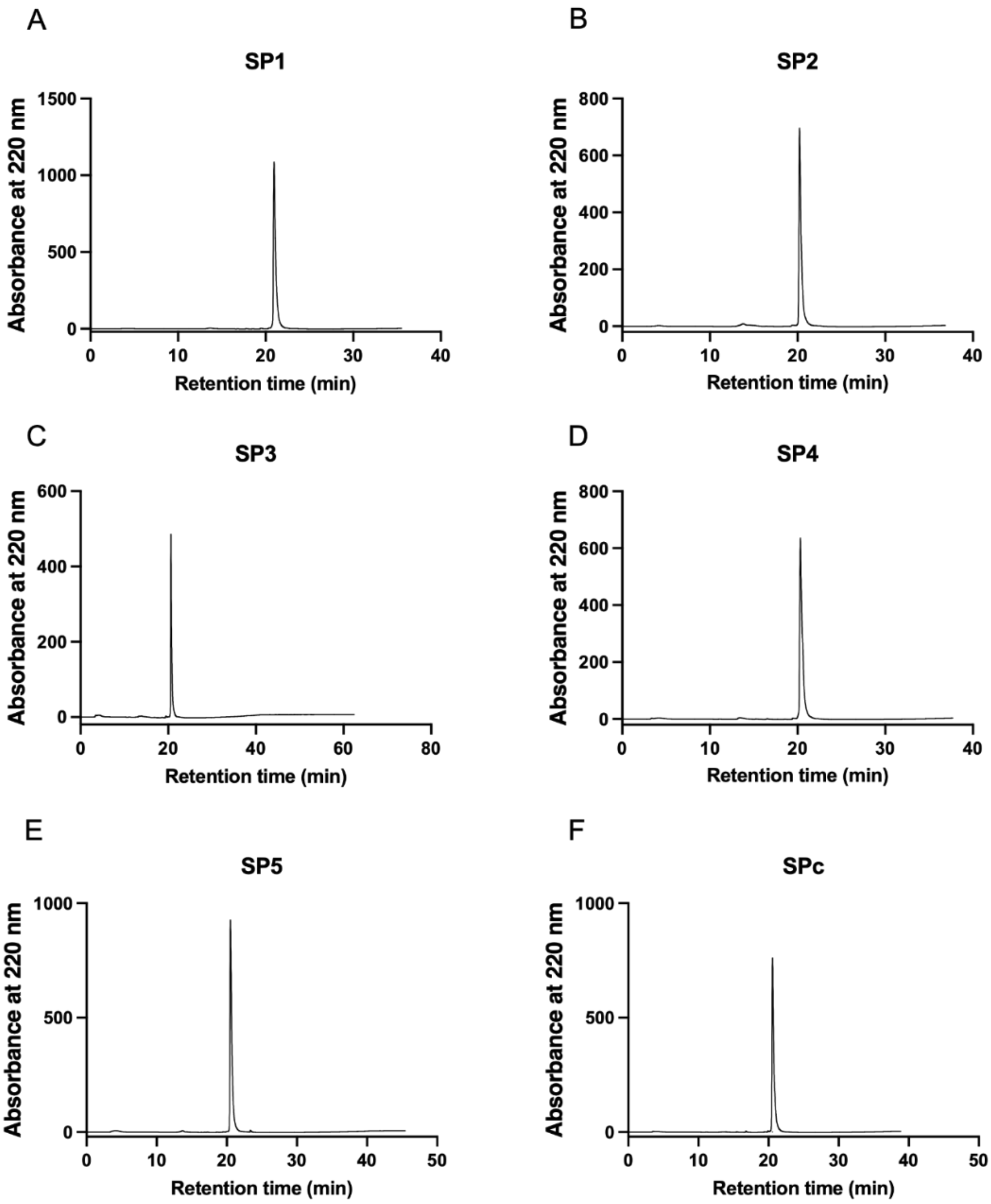

Figure S6. *RP-HPLC chromatograms of (**A**) SP1, (**B**) SP2, (**C**) SP3, (**D**) SP4, (**E**) SP5, and (**F**) SPc, recorded at 220 nm. All probes showed ≥95% purity by peak integration.*

Table S2. *Acetonitrile percentage of mobile phase at secondary probe elution during analytical RP-HPLC.*

**Secondary probe**	**% Acetonitrile at elution**
HP20	20.5
SP1	53.2
SP2	50.8
SP3	52.2
SP4	51.1
SP5	51.8
SP_C	51.9

MATERIALS AND METHODS

General

SKOV3 was obtained from the American Type Culture Collection (ATCC, Manassas, VA, USA) and cultured in RPMI 1640 (Biochrom GmbH, Berlin, Germany) supplemented with 10% fetal bovine serum (FBS) (Sigma Aldrich, St. Louis, MO, USA). Cells were grown in a humidified incubator at 37 °C in a 5% CO_2 atmosphere. Chemicals were purchased from Thermo Fisher Scientific (Waltham, MA, USA) or Merck (Darmstadt, Germany), except where otherwise indicated. No carrier added [^{177}Lu]$LuCl_3$ was purchased from ITM Isotope Technologies Munich (Munich, Germany). Radioactivity was measured using a dose calibrator RDC-VIK-202 (COMECER Netherlands, 8501-XC, Jourse, Netherlands) equipped with an ionizing chamber. For cell and animal studies, radioactivity was measured using a gamma-spectrometer with a NaI (Tl) detector (2480 Wizard, Wallac, Finland). A Cyclone Storage Phosphor System (CR-35 BIO Plus, Elysia-Raytest, Bietigheim-Bissingen, Germany) and OptiQuant image analysis software v.2.0 (PerkinElmer, Waltham, MA, USA) were used for measuring the radioactivity distribution on instant thin layer chromatography (iTLC) strips. NAP-5 (Cytiva, Uppsala, Sweden) columns were used for purification if necessary.

Biodistribution data were analyzed by one-way ANOVA with Bonferroni's multiple-comparison correction using GraphPad Prism (version 10 for Windows; GraphPad Software) to determine significant differences ($p < 0.05$). Biodistribution sample sizes were n = 3 (Figure 2, 3), with no excluded data. Data are presented as mean ± SD. SPR experiments were performed in duplicate and analyzed using a 1:1 binding model in Biacore T200 software. LigandTracer cell-binding experiments were analyzed using InteractionMap. Error bars represent SD unless otherwise specified.

Detailed Description of the Synthesis of PNA/peptide Probe Backbones

Both secondary and primary PNA probes were synthesized by a combination of manual and automated solid phase peptide synthesis (SPPS) using a Biotage Initiator+ Alstra on a 0.1 mmol scale in 10 mL reactor vials using the Rink Amide Tentagel XV RAM resin (0.27 mmol/g) purchased from Iris Biotech. The synthesis was performed using the 9-fluorenylmethyloxycarbonyl (Fmoc) synthesis strategy. Fmoc-protected amino acids (AAs) obtained from Novabiochem include Fmoc-Glu(OtBu)-OH, Fmoc-Tyr(tBu)-OH, Fmoc-Ser(tBu)-OH and Fmoc-Lys(Mmt)-OH. Fmoc-Gly-OH, Fmoc-8-amino-3,6-dioxaoctanoic acid (Fmoc-AEEA) and DOTA-tri(t-butyl ester) were purchased from AmBeed. Fmoc-Lys(Ac)-OH and Boc-L-Glu(OtBu)-OH were purchased from Combi-Blocks. Fmoc-Lys(Mtt)-

OH was obtained from aablocks, Fmoc-Cys(Trt)-OH from Merck and Fmoc-NH-PEG8-CH_2COOH from Angene.

The Fmoc protecting group on the resin and subsequent coupled residues was removed by washing the resin with 20% piperidine in dimethylformamide (DMF) once for 3 minutes, then two more times for 10 minutes each followed by two washes with DMF. Automated couplings (the entirety of HP9 and the sequences of the secondary probes up to the Cys residue) were synthesized using Oxyma Pure and diisopropylcarbodiimide (DIC) in DMF at 75°C for 10 min. All residues excluding PNAs were coupled with a six-equivalent excess at a final concentration of 0.2 M. PNA monomers were coupled in an identical way but in an excess of four equivalents, instead of six. All residues were dissolved in DMF except for the C monomer which was dissolved in *N*-methyl-2-pyrrolidone (NMP). Coupling reagents were added in equivalents matching the equivalents of the residue being coupled. Following the coupling step a capping step was performed using NMP:2,6-lutidine:acetic anhydride (89:6:5) for 2 min at room temperature followed by two washes with DMF.

After the coupling of Fmoc-Lys(Mtt)-OH as the second residue of the peptide (included in the C-terminal part of all probes, for attachment of the DOTA chelator), the side chain was manually and selectively Mtt-deprotected by repeated resin washes with trifluoroacetic acid (TFA):triisopropylsilane (TIS):dichloromethane (DCM) (1:5:94). Washes were performed until complete Mtt deprotection was considered, this by observing the wash solution turning yellow, then reverting to transparent. Following Mtt deprotection the resin was neutralized by washing once with 1% N-ethyldiisopropylamine (DIEA) in DMF. DOTA-tri(t-butyl ester) was selectively coupled to the Lys side chain using DIC and Oxyma Pure at 75°C in identical equivalents as for AAs. The last residues of the secondary probes following the coupling of Fmoc-Cys(Trt)-OH were coupled manually using 5 equivalents of AA, DIEA and (benzotriazol-1-yloxy)tripyrrolidinophosphonium hexafluorophosphate (PyBOP) in DMF at room temp.

Cleavage and Purification of Complete PNA/peptide Probes

Fully synthesized probes were initially dried by repeated washes with DCM followed by solvent evaporation. Probes were cleaved off the dried resin by dissolving the resin in TFA:TIS:Milli-Q:1,2-ethanedithiol (EDT) (92.5:2.5:2.5:2.5) and left on a shaker for 3 hours at room temp. The cleaving mix was separated from the resin and precipitated dropwise in a ten

times larger volume of cold diethyl ether. The precipitate was pelleted by centrifugation at 4000 rcf for 5 min and the remaining liquid poured off and separated from the pellet. The peptide pellet was then resuspended in new cold ether followed by another round of centrifugation and separation of ether liquid. This washing process was repeated for a total of three times before the pellet was left to dry by evaporation overnight. The dried pellet was re-dissolved in a 1:1 mix of 0.1% TFA in acetonitrile (ACN) and 0.1% TFA in Milli-Q, frozen, and freeze-dried overnight giving a crude PNA-peptide powder.

Purification of synthesized PNA probes was performed using reversed-phase high-performance liquid chromatography (RP-HPLC) on an Agilent Technologies 1260 Infinity 2 machine using a ZORBAX 300SB-C18 semi-preparative 9.4x250 mm 5-Micron column. Elution of injected sample was performed in a gradient setting starting at 10% buffer B (0.1% TFA in ACN) and 90% buffer A (0.1% TFA in Milli-Q), with buffer B increasing to 60% over 50 minutes at 60°C. Peaks at 260 nm were collected and analyzed by matrix-assisted laser desorption/ionization mass spectrometry time-of-flight (MS-MALDI TOF). Peaks containing the correct full-length peptide were pooled, frozen and freeze-dried overnight.

Purity Analysis of Probes by Analytical RP-HPLC

Probe purity was analyzed by analytical RP-HPLC using a ZORBAX 300SB-C18 analytical column with 3.5 μm particle size and a 4.6x150 mm sized column. Each probe was injected, and elution run at 70°C in buffer A with a gradient of 0-100% buffer B over 40 minutes.

Construction of Expression Plasmids, Protein Expression and Purification

To create mpET-45b-$Z_{HER2:2891}$-SR-H_6, the DNA segment encoding $Z_{HER2:2891}$-SR-H_6 was amplified from pAY430-His_6-Z_{2891}-Cys (kind gift from Dr Nilvebrant, KTH) and subcloned into a modified pET45b vector (mpET-45b;[5]). The plasmid contains a LPETG sortase A recognition site (SR) for sortase A ligation, followed by a His_6 affinity tag (H_6) on the C-terminal for immobilized metal affinity chromatography (IMAC) purification. The plasmid was sent for sequencing (Microsynth) to confirm the correct DNA sequence. SrtA 7+;[6] Protein expression and purification were done as described elsewhere[5]. The purity and mass of the proteins after IMAC purification were confirmed using MALDI-TOF MS.

Conjugation and Purification of PNA-Affibody Constructs

The conjugation of HP9 to $Z_{HER2:2891}$-SR-H_6 was essentially done as described previously[5]. Briefly, 660 nmol of HP9 was mixed with 440 nmol of $Z_{HER2:2891}$-SR-H_6 in 50 mM HEPES, 500 mM NaCl, pH 8.0. The conjugation reaction was initiated by adding SrtA 7+ and incubating the mixture for 20 min at 37 °C. The reaction mixture was then processed using a Ni-NTA HisPur (Thermo Scientific) column. Nonreacted and His_6-tagged affibody was allowed to bind to the resin, followed by elution increasing concentrations of imidazole. SDS-PAGE and RP-HPLC were used for monitoring and purification. The mass of $Z_{HER2:2891}$-HP9 conjugate was verified using MALDI TOF.

Structural Characterization Using Circular Dichroism Spectroscopy

All circular dichroism (CD) experiments were conducted using a CD spectrometer (Applied Photophysics, Leatherhead, UK) equipped with a Peltier thermoelectric controller and an external temperature probe inserted into the protein sample. A sample of 25 µM $Z_{HER2:2891}$-HP9 was prepared in 10 mM potassium phosphate buffer with 10 mM KCl at pH 7.4. CD measurements were performed in a capped quartz cuvette with a 0.1 cm path length. The unfolding of $Z_{HER2:2891}$-HP9 was monitored by measuring the ellipticity at 221 nm (θ_{221}) as a function of temperature. Temperature was increased from 20 to 100°C in 1°C increments at a speed of 1 °C/min. The melting temperature (T_m) was determined as the maximum of the first derivative of the melting curve using the Global 3 analysis software (Applied Photophysics, Leatherhead, UK). Additionally, CD spectra in the range of 195–300 nm were recorded for $Z_{HER2:2891}$-HP9 at 20°C before and after treatment at 100°C to monitor signal recovery. All CD spectra were baseline subtracted and presented as an average of five scans.

Surface Plasmon Resonance Based Binding analysis

We conducted Surface Plasmon Resonance (SPR) binding studies using a Biacore T200 instrument (Cytiva) at 25°C. All experiments utilized dextran-coated CM5 sensor chips with PBS-T buffer (pH 7.4). For primary binding analysis, recombinant human HER2/Fc chimera (Sino Biologicals) was immobilized on a CM5 chip via standard EDC/NHS coupling to achieve 660 resonance units (RU), followed by ethanolamine sealing. A reference surface without immobilized protein was prepared in parallel. Z-HP9 was injected at concentrations ranging from 0.8-25 nM (0.8, 1.6, 3.1, 6.3, 12.5, and 25 nM) at a flow rate of 50 µL/min. Association and dissociation phases were monitored for 300 and 100 seconds, respectively. Surface regeneration was achieved using two 30-second injections of 10 mM glycine-HCl (pH 1.5).

For secondary probe binding analysis, Z-HP9 was immobilized at 430 and 450 RU on two separate surfaces of a CM5 chip. DM1-labeled PNA-based probes (SP1-SP5 and SPc) were injected at concentrations of 1.4, 7, 14, and 70 nM with a flow rate of 30 µL/min. Association and dissociation were monitored for 700 and 1800 seconds, respectively. For comparison, Z-HP9 was immobilized at 200 RU on another CM5 chip surface, and HP20 was injected at concentrations of 2.2, 4.4, 8.8, 17.5, and 35 nM. Flow rates, association/dissociation times, and surface regeneration for this comparison followed the same protocol used for the DM1-labeled compounds. All data analysis was performed using Biacore T200 Evaluation software (version 2.0) with a 1:1 binding model.

References

(1) Wållberg, H., Orlova, A., Altai, M., Hosseinimehr, S.J., Widström, C., Malmberg, J., Ståhl, S., Tolmachev, V. (2011) Molecular design and optimization of 99mTc-labeled recombinant affibody molecules improves their biodistribution and imaging properties. J Nucl Med. 52, 461-9.

(2) Westerlund, K., Oroujeni, M., Gestin, M., Clinton, J., Hani Rosly, A, Tano, H., Vorobyeva, A., Orlova, A., Eriksson, K.A, Tolmachev, V. (2024) Shorter Peptide Nucleic Acid Probes Improve Affibody-Mediated Peptide Nucleic Acid-Based Pretargeting. ACS Pharmacol Transl Sci. 7, 1595-1611.

(3) Feldwisch, J., Tolmachev, V., Lendel, C., Herne, N., Sjöberg, A., Larsson, B., Rosik, D., Lindqvist, E., Fant, G., Höidén-Guthenberg, I., Galli, J., Jonasson, P., Abrahmsén, L. (2010) Design of an optimized scaffold for affibody molecules. J Mol Biol. 398, 232-47.

(4) Tano, H., Oroujeni, M., Vorobyeva, A., Westerlund, K., Liu, Y., Xu, T., Vasconcelos, D., Orlova, A., Karlström, A.E., Tolmachev, V. (2021) Comparative Evaluation of Novel ^{177}Lu-Labeled PNA Probes for Affibody-Mediated PNA-Based Pretargeting. Cancers (Basel). 13, 500.

(5) Altai, M., Westerlund, K., Velletta, J., Mitran, B., Honarvar, H., Karlström, A.E. (2017) Evaluation of affibody molecule-based PNA-mediated radionuclide pretargeting: Development of an optimized conjugation protocol and ^{177}Lu labeling. Nucl Med Biol. 54, 1-9

(6) Jeong, H.J., Abhiraman, G.C., Story, C.M., Ingram, J.R., Dougan, S.K. (2017) Generation of Ca2+-independent sortase A mutants with enhanced activity for protein and cell surface labeling. PLoS One.12, e0189068.
